# From arthritis to central sensitization: targeting the neuro-immune axis via microglial modulation in acupuncture treatment for Juvenile Idiopathic Arthritis–associated pain

**DOI:** 10.3389/fimmu.2025.1684060

**Published:** 2026-01-09

**Authors:** Hang Chen, Jixiang Xu, Xia Zhang

**Affiliations:** 1 Department of Pediatrics, The First Affiliated Hospital, Henan University of Chinese Medicine, Zhengzhou, Henan, China; 2 School of Pediatrics, Henan University of Chinese Medicine, Zhengzhou, Henan, China

**Keywords:** Juvenile Idiopathic Arthritis (JIA), chronic pain, central sensitization, microglia, acupuncture, neuro-immune axis, vagus nerve, mitochondria

## Abstract

Juvenile Idiopathic Arthritis (JIA) is a common chronic rheumatic disease in children, and its associated persistent pain severely impacts the quality of life of affected patients. The severity of JIA-associated pain often does not correlate with the degree of peripheral inflammation, suggesting that Central Sensitization is a key underlying mechanism specific to JIA subtypes. At the core of this process is a dysregulation of the neuro-immune axis, particularly the aberrant activation of microglia within the central nervous system. As a non-pharmacological therapy, acupuncture demonstrates significant potential for JIA pain management, supported by a series of clinical studies. This review focuses on JIA-specific neuro-immune pathophysiology of chronic pain and systematically elucidates the complete pathway from peripheral inflammation to central microglial activation and central sensitization. By integrating evidence from both preclinical and clinical JIA studies, we provide a detailed analysis of how acupuncture remodels neuro-immune balance through mechanisms spanning peripheral autonomic regulation and central glial modulation. This modulation encompasses the inhibition of upstream pro-inflammatory signaling pathways. These include peripheral anti-inflammatory effects via the vagus nerve, regulation of systemic immunity, and direct inhibition of central microglial activity toward an anti-inflammatory phenotype. This work establishes a theoretical framework, grounded in JIA-specific neuro-immune pathophysiology, for acupuncture’s application in treating JIA-associated pain, highlighting its role in reducing reliance on analgesics.

## 1 Introduction

Juvenile Idiopathic Arthritis (JIA) is the most common chronic rheumatic disease of childhood (1). It not only causes joint damage and functional disability but also severely impacts the quality of life of affected children through persistent pain, posing a long-term and profound threat to their physical development, psychological well-being, and social functioning (2). Chronic pain is extremely prevalent among children with JIA; it is not merely a physiological symptom but a complex condition highly co-morbid with anxiety, depression, and sleep disturbances, which significantly reduces health-related quality of life (HRQoL) (3) Although disease-modifying antirheumatic drugs (DMARDs) and biologic agents can effectively control peripheral joint inflammation in most children, a formidable clinical challenge has emerged: a substantial portion of patients do not experience complete pain relief even as inflammatory markers improve. The pain may even persist during periods of clinical “remission” of the arthritis. This “disconnect” between pain and the degree of peripheral inflammation strongly suggests the involvement of more complex pathological mechanisms that extend beyond the peripheral joints themselves (4).

To explain this clinical dilemma, the theory of Central Sensitization has become a core framework for understanding JIA and other chronic pain states (5). Central Sensitization is defined as a state of maladaptive plasticity in the central nervous system (specifically the spinal cord and brain) following persistent or intense noxious input. This process involves functional and structural remodeling of neurons, leading to abnormally enhanced excitability and weakened inhibitory function. Ultimately, this results in an amplified and distorted processing of both noxious and non-noxious stimuli (5). This process serves as the neurobiological basis for hyperalgesia (exaggerated pain from a painful stimulus) and allodynia (pain from a normally non-painful stimulus), providing a scientific explanation for why children with JIA may experience pain from a simple touch and successfully shifting the research focus from the peripheral joints to the central nervous system (4).

The biological foundation of central sensitization is rooted in the profound dysregulation of the Neuro-Immune Axis, within which microglia—the resident immune cells of the central nervous system—play a critical role (6) In the pathogenesis of JIA, inflammatory cytokines released from the peripheral joints, such as Tumor Necrosis Factor-alpha (TNF-α) and Interleukin-1beta (IL-1β), can transmit “alarm” signals to the central nervous system via humoral or neural pathways (7, 8). These signals can activate microglia from their quiescent, surveying state, inducing a transformation into an activated, pro-inflammatory M1 phenotype. Activated microglia then release a variety of mediators, including Brain-Derived Neurotrophic Factor (BDNF), which directly modulate neuronal synaptic plasticity, acting as a central driver that initiates and sustains central sensitization (8, 9). Furthermore, gut microbiota dysbiosis has been implicated in JIA pathogenesis, altering immune responses that may contribute to neuro-immune dysregulation (10).

In this context, the development of non-pharmacological therapies that can effectively intervene in central sensitization and are safe for pediatric use has emerged as a clinical and research priority. The long-term use of conventional analgesic drugs, including non-steroidal anti-inflammatory drugs (NSAIDs) and opioids, is limited in children due to risks such as gastrointestinal and renal damage, as well as potential dependence (11). Acupuncture, a physical therapy with millennia of history, has garnered significant attention for its remarkable efficacy and high safety profile in chronic pain management, particularly in JIA (12). Modern research has confirmed that the effects of acupuncture are not merely a placebo response but are based on profound regulation of the neuro-immune axis across multiple systems and targets (13, 14).

The focus of this paper is to attempt a systematic integration of four relevant scientific fields: pediatric rheumatology, pain neuroscience, cellular neuroimmunology, and evidence-based complementary medicine. Our goal is to delineate and construct an integrative mechanistic model, aiming to elucidate the potential mechanisms by which this therapy addresses the modern medical challenge of JIA-associated pain. Its significance is twofold. At the level of basic research, it provides a cellular and molecular explanation for acupuncture’s analgesic effects centered on microglia, opening new research perspectives in neuroimmunology. At the level of clinical application, it offers a solid mechanistic rationale for incorporating acupuncture into the comprehensive management of JIA, potentially promoting a safer, more holistic model for pediatric chronic pain management and reducing reliance on conventional analgesics.

**Figure 1:**
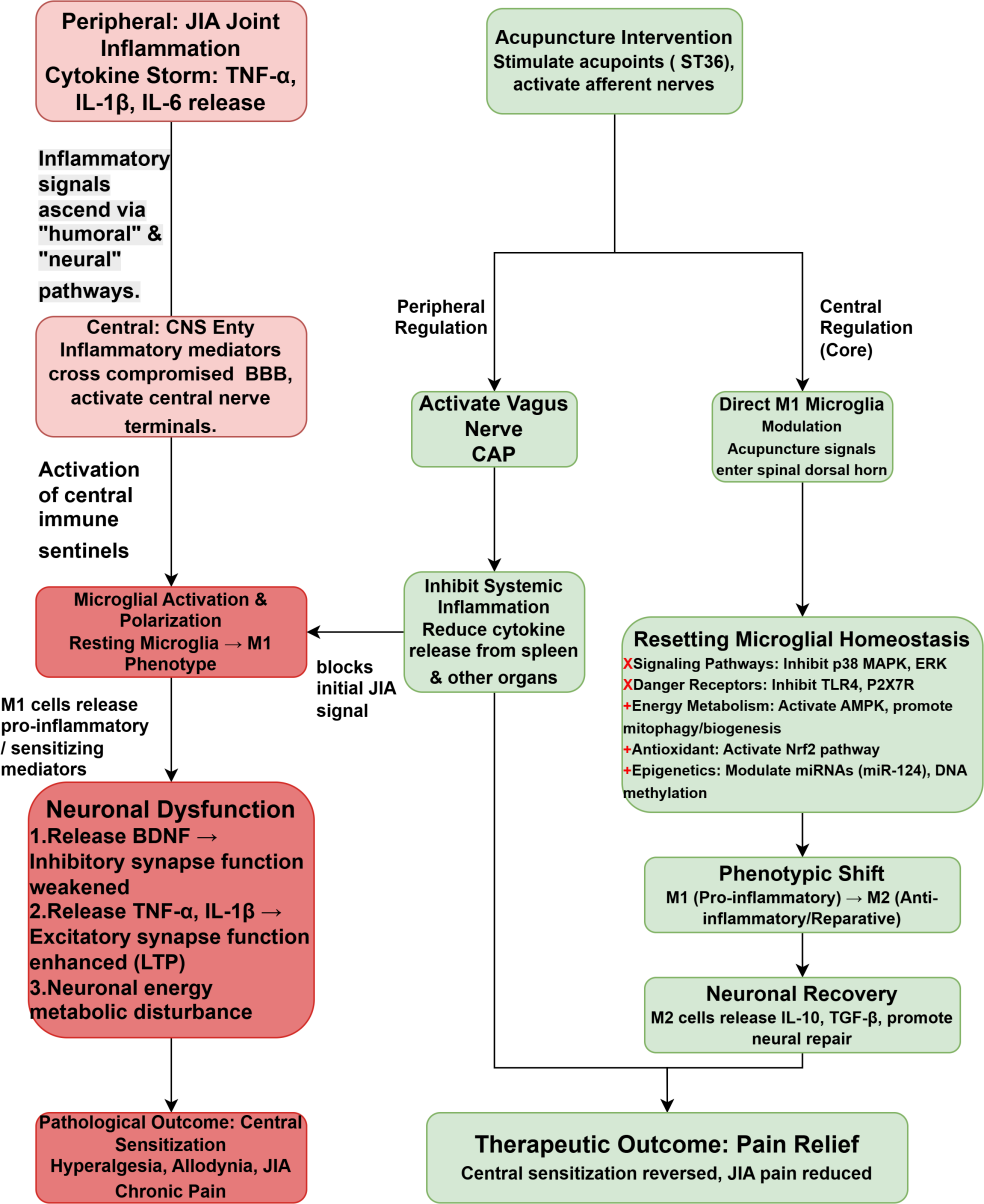
Flowchart of the neuro-immune pathological mechanism of JIA-Associated pain and the multi-pathway intervention of acupuncture.

Therefore, the specific objectives of this paper are: (1) To review and delineate the complete pathophysiological chain, from peripheral joint inflammation in JIA to central microglial activation and the subsequent formation of central sensitization; (2) To critically analyze and synthesize existing preclinical and clinical evidence to clarify the modulatory effects of acupuncture on the neuro-immune axis at multiple peripheral and central levels; and (3) To ultimately construct a logically rigorous biological model, with microglia as the key target, to explain the intrinsic mechanisms of acupuncture in treating JIA-associated pain, thereby identifying future directions for in-depth research and clinical translation. The core pathological and therapeutic pathways discussed in this review are summarized in **Figure 1**.

## 2 Literature search strategy

The literature serving as the basis for this descriptive review was identified through a comprehensive search of major electronic databases, including PubMed, Web of Science, Embase, and the Cochrane Library, for articles published up to August 2025.Key search terms were used in combination to reflect the core concepts of this paper, including: (“Juvenile Idiopathic Arthritis” OR “JIA”) AND (“central sensitization” OR “microglia” OR “neuro-immune axis”) AND (“acupuncture” OR “electroacupuncture” OR “non-pharmacological pain management”).The selection of literature was guided by its relevance to the review’s primary objectives: elucidating the neuro-immune pathophysiology of JIA-associated pain and the molecular mechanisms of acupuncture intervention. Peer-reviewed preclinical and clinical studies addressing these specific topics were prioritized. Articles not published in English or those unrelated to the central neuro-immune pathways of pain were excluded from the analysis.

## 3 Neuro-immune pathophysiology of JIA pain: peripheral inflammation and central sensitization

This chapter aims to provide an in-depth analysis of the complete process by which JIA pain evolves from a localized joint issue into a central nervous system phenomenon.

### 3.1 Pathophysiological basis of JIA and the inflammatory cascade

JIA is a complex autoimmune disease driven by a combination of genetic susceptibility, environmental triggers, and immune system dysregulation. Its pathophysiology begins with a predisposing genetic background. Genome-Wide Association Studies (GWAS) have identified multiple susceptibility loci associated with JIA, the most significant of which is the Human Leukocyte Antigen (HLA) region. Specific alleles of HLA class II molecules, such as HLA-DRB1 and HLA-DPB1, play a critical role in antigen presentation, determining whether self-peptides can be effectively presented to T cells, thereby initiating an autoimmune response (15, 16). Furthermore, variations in non-HLA genes like PTPN22, STAT4, and IL2RA are also linked to JIA risk. The proteins encoded by these genes are often key regulators in immune signaling pathways, and their dysfunction can lead to a breakdown of immune tolerance (17, 18).

Building on this genetic foundation, the aberrant recognition of and response to self-antigens constitutes the initiating autoimmune cascade of JIA. This process is hallmarked by the production of various autoantibodies, such as Antinuclear Antibodies (ANA) and Rheumatoid Factor (RF), which serve as important diagnostic and prognostic markers for specific JIA subtypes. Although the direct pathogenic role of these antibodies is still under investigation, their presence clearly indicates aberrant B cell activation and a loss of immune tolerance. Within the articular synovium, the primary site of pathology, antigen-presenting cells (APCs) capture and present self-antigens, activating CD4+ T cells and promoting their differentiation into pro-inflammatory helper T cell subsets, predominantly Th1 and Th17 cells (19). Th1 cells secrete Interferon-gamma (IFN-γ), while Th17 cells produce cytokines, most notably IL-17. These two cell types act synergistically, weaving a potent pro-inflammatory network (19).

The core effectors of this network are located in the synovial tissue. Driven by a cytokine storm, the synovium undergoes dramatic changes: resident macrophages and fibroblast-like synoviocytes (FLS) are activated and proliferate extensively, forming an invasive tissue layer known as the pannus (20). These activated FLS exhibit “tumor-like” behavior; they produce large quantities of matrix metalloproteinases (MMPs), such as MMP-1 and MMP-3, which directly degrade the cartilage matrix (20). Cytokines like TNF-α and IL-1β induce endothelial cells in the synovial blood vessels to upregulate adhesion molecules such as ICAM-1 and VCAM-1, facilitating the transmigration of immune cells like neutrophils and lymphocytes from the circulation into the synovial tissue, further amplifying the inflammatory response (21). Activated T cells and FLS highly express receptor activator of nuclear factor kappa-B ligand (RANKL), which binds to RANK on osteoclast precursors, inducing their differentiation and leading to bone destruction and erosion at the articular margins (20).

The vast array of pro-inflammatory mediators generated by this cascade—including TNF-α, IL-1β, IL-6, and various chemokines—not only causes local joint swelling, pain, and structural damage but also acts as “molecular messengers.” They transmit inflammatory signals over long distances to the central nervous system, either via the circulatory system or by directly activating afferent nerve fibers, thereby setting the stage for the subsequent initiation of central sensitization. Therefore, the joint pathology in JIA is not an isolated local problem but rather the “epicenter” that initiates a systemic chain reaction, particularly one that extends to the central nervous system (22).

### 3.2 Neural transmission of inflammatory signals and central sensitization mechanisms

The initial transmission of pain signals from the peripheral joints to the central nervous system (CNS) occurs via Aδ and C nociceptive fibers, but chronification involves a long-distance relay orchestrated by inflammatory mediators (23). This process begins with peripheral sensitization, wherein high concentrations of pro-inflammatory mediators in the synovial tissue—such as TNF-α, IL-1β, and nerve growth factor (NGF)—act directly on the terminals of nociceptive sensory neurons (24). Through mechanisms like phosphorylation of ion channels (e.g., TRPV1) and alterations in gene expression, these mediators significantly lower the activation threshold of the neurons, causing them to fire abnormally even in response to normal stimuli (22, 25). These “ignited” neurons then transmit a relentless barrage of noxious signals along their axons to the dorsal horn of the spinal cord.

Once these signals enter the spinal cord dorsal horn, they trigger the pathological cascade of central sensitization. This process critically involves the activation of spinal glial cells, particularly microglia and astrocytes. Inflammatory cytokines in the circulation can access the CNS via two primary routes: (1) across a compromised blood-brain barrier (BBB), where persistent systemic inflammation disrupts tight junction proteins (e.g., Claudin-5, Occludin) via TNF-α and IL-1β, increasing permeability; (2) via neural pathways, where nociceptive sensory neurons transmit electrical signals to the CNS but modulate the inflammatory milieu by releasing neuropeptides such as Substance P and calcitonin gene-related peptide (CGRP) from their central terminals, directly activating glial cells (23, 25).

Once activated, glial cells transform from homeostatic “guardians” into inflammatory “amplifiers.” A vicious positive feedback loop is established between activated microglia and astrocytes. They sense danger signals via surface pattern recognition receptors (e.g., TLR4) and release a second wave of pro-inflammatory mediators, including TNF-α, IL-1β, IL-6, and nitric oxide (NO) (26, 27). These mediators further enhance the excitability of dorsal horn projection neurons. The molecular mechanisms underlying this process involve enhancement of excitatory synaptic transmission—glutamate and ATP released by glia act on presynaptic and postsynaptic NMDA and AMPA receptors, inducing long-term potentiation (LTP), a key mechanism of synaptic plasticity underlying pain “memory” —and weakening of inhibitory synaptic transmission, where activated microglia release BDNF, downregulating the KCC2 chloride co-transporter and impairing GABAergic interneurons (8, 28).

Ultimately, this glia-driven neuroinflammation leads to lasting functional and structural remodeling of neurons in the spinal dorsal horn and higher brain regions (such as the thalamus and anterior cingulate cortex). Neurons become hypersensitive to input signals, and their receptive fields expand, causing pain to be amplified in intensity and to spread spatially. This is the fundamental reason for the hyperalgesia and allodynia experienced by children with JIA, marking the transition of pain from a symptom to a “disease state” that is self-sustained by the CNS itself (28).

### 3.3 Microglial activation and functional polarization

Microglia are the sentinels and first line of defense of the CNS, and the switch in their functional state is a central event in neuroinflammation and the chronification of pain. Under homeostatic conditions, microglia exhibit a ramified morphology, constantly extending their processes to survey the microenvironment. Upon sensing inflammatory signals from the periphery or internal danger-associated molecular patterns (DAMPs), microglia are rapidly activated, transforming their morphology from ramified to amoeboid and initiating a range of functional programs (29–31).

Activated microglia display remarkable functional plasticity, traditionally simplified into two opposing polarization phenotypes: M1 (classical activation) and M2 (alternative activation). The M1 phenotype is considered “pro-inflammatory” and “neurotoxic.” Stimulated by signals like IFN-γ and lipopolysaccharide (LPS), M1 cells—via the activation of transcription factors like NF-κB and STAT1—produce and release large amounts of pro-inflammatory cytokines (TNF-α, IL-1β, IL-6), reactive oxygen species (ROS), and nitric oxide (NO). Together, these molecules exacerbate neuronal injury and drive pain sensitization (26, 32). In models of JIA-related chronic pain, the persistent presence of M1-polarized microglia in the spinal dorsal horn is a key factor in maintaining central sensitization (33).

In contrast, the M2 phenotype serves “anti-inflammatory” and “neuroprotective” functions. Induced by cytokines such as IL-4 and IL-13, M2 cells—via activation of the STAT6 signaling pathway—upregulate the expression of arginase-1 (Arg-1), produce anti-inflammatory cytokines like IL-10 and transforming growth factor-beta (TGF-β), and release various neurotrophic factors, including BDNF, to suppress inflammation, clear cellular debris, and promote tissue repair (9, 34). One pivotal study demonstrated that IL-4 could induce a specific CD11c+ M2-like microglial subset that played a crucial role in resolving neuropathic pain, highlighting the direct function of M2 polarization in pain resolution (9).

However, the M1/M2 model is a simplified framework. The evidence indicates that microglial functional states exist on a continuum and are diverse, influenced by context-specific signals in chronic pain (35). The TREM2 (triggering receptor expressed on myeloid cells 2) signaling pathway plays a key regulatory role in this process. TREM2 is an important receptor on the microglial surface that can sense various ligands, including lipids and apoptotic cells. Its signaling is crucial for microglial survival, proliferation, phagocytosis, and the transition from a homeostatic state to a disease-associated microglia (DAM) phenotype (36). In models of neurodegenerative disease, TREM2 deficiency impairs the ability of microglia to clear pathological proteins (like amyloid-beta) and exacerbates neuroinflammation. In the context of chronic pain, dysregulation of the TREM2 pathway may prevent microglia from effectively clearing inflammatory debris and repairing damage, thereby prolonging the pro-inflammatory M1 state. Therefore, the core pathology of pain chronification in JIA may lie not only in the over-activation of M1 microglia but also in the failure of their transition to the M2 restorative state, linked to an impairment in key regulatory functions mediated by TREM2 (36).

### 3.4 Maladaptive remodeling and impaired repair of neural networks

The chronic pain associated with JIA is, in essence, a form of maladaptive neuroplasticity (26). Persistent neuroinflammation not only alters neuronal excitability but also leads to durable remodeling of the structure and function of the central nervous system. In pain-related regions of the spinal cord and brain, changes occur in the density and morphology of neuronal dendritic spines; new, aberrant synaptic connections are formed, while existing normal connections may be weakened or eliminated (8). This structural reorganization provides the physical basis for the consolidation and long-term maintenance of pain “memory.” Furthermore, the chronic inflammatory environment can impair mitochondria, the core of neuronal energy metabolism. Mitochondrial dysfunction—manifesting as reduced ATP production, excessive generation of reactive oxygen species (ROS), and the release of mitochondrial DNA (mtDNA)—not only directly harms neurons but also further exacerbates neuroinflammation and neuronal death by activating the cGAS-STING signaling pathway and necroptosis pathway (37, 38).

Beyond the neurons themselves, damage to the myelin sheath and impairments in its repair represent a frequently overlooked but critical component of chronic pain pathology in JIA models (39). The myelin sheath is a lipid-rich insulating layer that wraps around nerve axons, essential for the rapid and efficient saltatory conduction of nerve signals. In a sustained neuroinflammatory environment, oligodendrocytes—the cells responsible for producing and maintaining myelin in the CNS—can become dysfunctional or even die, leading to demyelination. Demyelination not only slows nerve conduction but also exposes potassium channels on the axonal membrane, leading to an abnormal resting potential and spontaneous neuronal firing, directly generating ectopic pain signals (40, 41).

Worse still, the debris generated from myelin damage is itself a potent inflammatory stimulus and an inhibitor of regeneration. This myelin debris must be promptly cleared by phagocytic cells, such as microglia and macrophages, to create a permissive environment for the differentiation of oligodendrocyte precursor cells (OPCs) and subsequent remyelination (42, 43). However, in a state of chronic inflammation, the phagocytic function of microglia is often impaired, leading to the accumulation of myelin debris, which further inhibits axonal regeneration and functional recovery (43). Therefore, the chronic pain associated with JIA is not merely a “software” problem (neuronal function) but also one of “hardware” damage (neural circuits and myelin structure). Any effective therapeutic strategy must simultaneously consider how to suppress neuroinflammation, promote neuroprotection, and ultimately achieve the repair and functional remodeling of both neural networks and myelin structures (44).

## 4 The multi-level therapeutic mechanisms of acupuncture: from somatosensory input to neuro-immune output

Acupuncture, as a classic physical therapy, is fundamentally a precise process of neuromodulation. The generation of its therapeutic effects is rooted in a core mechanism that can be analyzed by modern neuroscience: the somatosensory-autonomic reflex. This reflex arc provides the conceptual framework for this chapter, illustrating how a localized physical stimulus (needling) is converted into a systemic, multi-system biological regulation. Specifically, this process includes signal input via stimulation of Aδ and C-type sensory nerve fibers at acupoints; central integration in the brainstem (e.g., nucleus tractus solitarius) and higher centers (e.g., thalamus, hypothalamus); signal output through the autonomic nervous system (ANS) and hypothalamic-pituitary-adrenal (HPA) axis; and effector regulation of immune cells in target organs and CNS neuroinflammation (13, 45). The effects of acupuncture exhibit parameter dependency; efficacy varies with frequency, intensity, and acupoint selection, confirming its basis as neuromodulation (46). For pediatric JIA patients, non-invasive methods like laser acupuncture enhance acceptability while activating this pathway (47).

### 4.1 The core efferent pathway: the vagus nerve-cholinergic anti-inflammatory pathway

The vagus nerve-cholinergic anti-inflammatory pathway (CAP) is a key efferent route in the somatosensory-autonomic reflex, mediating acupuncture’s systemic anti-inflammatory effects. Stimulation of acupoints like Zusanli (ST36) activates vagal efferents, releasing acetylcholine (ACh) in immune organs such as the spleen (48). ACh binds to α7 nicotinic acetylcholine receptors (α7nAChR) on cholinergic T cells, which relay signals to macrophages (49).

Binding of ACh to α7nAChR on macrophages triggers anti-inflammatory cascades: (1) inhibition of NF-κB by blocking IκBα degradation, preventing transcription of TNF-α, IL-1β, and IL-6 (50); (2) suppression of NLRP3 inflammasome via JAK2/STAT3, blocking IL-1β maturation (51). Vagus activation also enhances BBB integrity, limiting peripheral inflammation’s CNS entry.

### 4.2 Central target regulation: reshaping microglial polarization state

Direct modulation of CNS microglia represents acupuncture’s core strategy for reversing central sensitization in JIA. Acupuncture shifts microglia from pro-inflammatory M1 to anti-inflammatory M2 phenotypes by resetting metabolic and redox homeostasis (52).

Key pathways include activation of AMP-activated protein kinase (AMPK) to inhibit NF-κB/p38 MAPK, promote mitophagy, and restore energy balance; and nuclear factor E2-related factor 2 (Nrf2) translocation to upregulate antioxidants like HO-1, clearing ROS and favoring M2 polarization. These actions suppress pro-inflammatory mediators (TNF-α, IL-1β) while promoting IL-10, TGF-β, and neurotrophins like BDNF/GDNF, altering central sensitization’s basis and enabling neuronal repair (53, 54).

## 5 Analysis of core mechanisms: the molecular basis of microglial modulation by acupuncture

Acupuncture’s core intervention in central sensitization lies in its capacity to effectively suppress the activation of spinal dorsal horn microglia from a resting state to a pro-inflammatory M1 phenotype. This suppression is achieved through the synergistic action of multiple pathways, which simultaneously block the input of upstream “danger signals” and cut off the downstream amplification of inflammatory signals.

### 5.1 Suppressing the “pro-inflammatory” program of microglia: synergistic action of multiple pathways

At the signal input level, acupuncture significantly downregulates the expression and function of several key pattern recognition receptors (PRRs) on the microglial surface. These receptors act as “antennas” through which microglia sense danger signals in their environment. Among them, Toll-like receptor 4 (TLR4) serves as the key receptor for recognizing bacterial lipopolysaccharide and endogenous damage-associated molecular patterns (DAMPs, such as high mobility group box 1, HMGB1), initiating the microglial pro-inflammatory response. Research has confirmed that electroacupuncture can block the influx of inflammatory signals at the source by downregulating the expression of TLR4 and its downstream adaptor protein, MyD88 (55, 56). Similarly, the P2X7 receptor (P2X7R), an ion channel-linked receptor activated by extracellular ATP, leads to calcium influx and direct activation of the NLRP3 inflammasome during tissue injury when high concentrations of ATP are released. Acupuncture has been shown to inhibit the over-activation of P2X7R, thereby reducing inflammasome activation and IL-1β release, effectively quenching the inflammatory fire driven by ATP (57, 58).

Downstream in the signal transduction cascade, acupuncture effectively inhibits key intracellular signaling pathways, particularly the p38 and extracellular signal-regulated kinase (ERK) pathways within the mitogen-activated protein kinase (MAPK) family (59, 60). The sustained phosphorylation and activation of p38 MAPK and ERK are core events driving M1 polarization in microglia (59, 60). They act as signal relay stations, transmitting signals from upstream receptors to the nucleus, ultimately activating key transcription factors like nuclear factor-kappa B (NF-κB) and initiating the expression of a range of pro-inflammatory genes (e.g., TNF-α, IL-1β, IL-6) (61). Multiple studies have demonstrated that electroacupuncture significantly inhibits the phosphorylation levels of p38 and ERK in spinal microglia in models of neuropathic pain, including JIA-like arthritis. By precisely inhibiting the activity of these MAPK pathway members, acupuncture effectively severs the pro-inflammatory signal flow from the cell membrane to the nucleus, thereby preventing the “pro-inflammatory program” of microglia from being initiated and maintaining an immune-quiescent state in the central nervous system (61).

### 5.2 Resetting cellular metabolism and oxidative stress: homeostatic regulation of mitochondrial function

Mitochondrial dysfunction is not only a hallmark of cellular energy failure but also a core engine driving microglial M1 polarization and the persistence of neuroinflammation. In chronic pain pathology (62), such as in JIA models, mitochondrial function within microglia becomes dysregulated, manifesting as reduced ATP production, excessive generation of reactive oxygen species (ROS), and the release of mitochondrial DNA (mtDNA) into the cytosol. These factors collectively activate the NLRP3 inflammasome, exacerbating the inflammatory response (63, 64). Acupuncture’s modulation of microglia is profoundly reflected in its ability to “reset” the mitochondrial quality control system, achieved primarily through a dual mechanism of clearing damaged mitochondria and promoting the generation of new, healthy ones.

Acupuncture promotes mitophagy—the selective clearance of damaged mitochondria—primarily by activating the PINK1/Parkin signaling pathway. In neuroinflammation, this process is often impaired, leading to the accumulation of dysfunctional mitochondria. The PINK1 protein accumulates on the outer membrane of damaged mitochondria, acting as a “distress signal” that recruits the Parkin ubiquitin ligase. Parkin then ubiquitinates the mitochondrion, tagging it for engulfment and degradation by autophagosomes. By efficiently clearing these “dysfunctional” organelles, acupuncture removes an internal source of persistent ROS production and inflammatory signals, thereby breaking the vicious cycle of inflammation (65, 66). Concurrently, while clearing old, damaged mitochondria, acupuncture promotes the generation of new ones by upregulating the expression and activity of peroxisome proliferator-activated receptor gamma coactivator 1-alpha (PGC-1α). PGC-1α is the “master transcriptional regulator” of mitochondrial biogenesis. Its activation initiates the expression of a series of downstream genes (such as NRF1 and TFAM), which in turn drives the creation of new mitochondria. This not only replenishes the cell’s energy reserves (ATP) but also enhances its overall capacity to combat oxidative stress (67, 68).

Through this elegant dynamic regulation of “out with the old, in with the new,” acupuncture restores the metabolic homeostasis of microglia, laying a solid bioenergetic foundation for their transition from a pro-inflammatory M1 phenotype to a neuroprotective and reparative M2 phenotype.

### 5.3 Regulating the “long-term memory” of gene expression: epigenetic modifications

The effects of acupuncture are not limited to immediate signal pathway inhibition; they also extend to producing more durable analgesic and anti-inflammatory outcomes by reprogramming the gene expression patterns of microglia through epigenetic modifications (69, 70). This regulation is primarily achieved through alterations in DNA methylation, histone modifications, and non-coding RNA expression, providing a molecular explanation for acupuncture’s sustained benefits in JIA-associated pain (69, 71).

Acupuncture influences the activity of DNA methyltransferases (DNMTs), such as DNMT1, to alter the methylation status of specific gene promoter regions, thereby achieving long-term regulation of their transcription. For instance, by inhibiting DNMT1 activity, acupuncture can demethylate promoters of genes involved in inhibitory neurotransmission, such as GABRD (encoding a GABA_A receptor subunit), restoring central inhibitory signaling and counteracting central sensitization (71). Histone modifications further contribute by regulating chromatin accessibility. Acupuncture balances histone acetyltransferases (HATs) and histone deacetylases (HDACs); specifically, by inhibiting HDACs, it increases histone acetylation levels in promoter regions, leading to a more relaxed chromatin structure. This promotes the expression of anti-inflammatory genes (like IL-10) while suppressing pro-inflammatory ones (71).

Non-coding RNAs, especially microRNAs (miRNAs), serve as fine-tuners of gene expression. Acupuncture regulates the expression of various miRNAs with anti-inflammatory functions. For example, electroacupuncture has been confirmed to upregulate miR-124 in the spinal cord. These miRNAs act as precise “molecular brakes” that can directly target and degrade the mRNA of key proteins in pro-inflammatory pathways (such as TRAF6 and IRAK1) or inhibit markers of microglial activation (72). More recent research has found that miR-26a, enriched in extracellular vesicles derived from neural stem cells, can suppress the pro-inflammatory polarization of microglia by targeting and inhibiting HMGA2 (73). This mechanism of precisely “switching off” the inflammatory response at the post-transcriptional level via miRNAs provides a powerful molecular explanation for the long-lasting effects of acupuncture.

These multi-level epigenetic regulatory mechanisms collectively form the molecular basis for the sustained effects of acupuncture, enabling it not only to alleviate symptoms but also to fundamentally reshape the immune microenvironment of the nervous system in JIA pain models.

This flowchart illustrates the pathological progression of JIA-associated chronic pain from the periphery to the central nervous system (Part 1, red path) and the core logic of acupuncture intervention (Part 2, green path).

Pathogenesis: Inflammatory mediators (e.g., TNF-α, IL-1β) released from JIA joint inflammation enter the Central Nervous System (CNS) via humoral or neural pathways. These signals activate spinal microglia, polarizing them toward the pro-inflammatory M1 phenotype. M1 microglia release mediators such as BDNF, TNF-α, and IL-1β, which enhance excitatory synaptic transmission (LTP) and weaken inhibitory synapse function. This drives neuronal dysfunction, ultimately leading to central sensitization and chronic pain.

Acupuncture Intervention: Acupuncture (e.g., stimulating ST36) activates afferent nerves to reset this dysregulated neuro-immune network via two primary pathways: (1) Peripheral Pathway: Activates the Vagus Nerve-Cholinergic Anti-inflammatory Pathway (CAP) to systemically inhibit peripheral inflammation (e.g., in the spleen), reducing the upstream inflammatory signaling. (2) Central Pathway: Acupuncture signals directly modulate spinal M1 microglia by inhibiting danger signal receptors (e.g., TLR4, P2X7R) and downstream pro-inflammatory pathways (e.g., p38 MAPK, ERK), while simultaneously activating energy homeostasis (AMPK), antioxidant pathways (Nrf2), and epigenetic modulation (e.g., miRNAs).

Therapeutic Outcome: This multi-target intervention collectively promotes a phenotypic shift in microglia from M1 (pro-inflammatory) to M2 (anti-inflammatory/reparative). M2 cells release anti-inflammatory factors like IL-10 and TGF-β, restoring neuronal homeostasis, and ultimately reversing central sensitization to alleviate JIA-associated pain.

## 6 Discussion and outlook

### 6.1 An integrative neuro-immune model of JIA pain and the interventional logic of acupuncture

Through a systematic review of the literature, this paper has constructed an integrative neuro-immune model of JIA pain that spans from the periphery to the central nervous system and from the macroscopic to the microscopic level. This model clearly posits that the persistent pain in JIA is not an isolated problem of joint inflammation but rather a dynamic process of dysregulation involving multiple systems. Its core chain of events is as follows: inflammatory signals from the peripheral joints are transmitted to the central nervous system via humoral and neural pathways, activating microglia, which act as “central immune sentinels.” Activated microglia, by releasing a vast array of pro-inflammatory mediators and altering their communication with neurons, drive and sustain maladaptive neuroplasticity in the spinal cord and brain, ultimately leading to central sensitization. In this model, microglia are not merely passive signal recipients but are “central nodes” for information processing and amplification (26, 74). Their functional state—specifically the imbalance between pro-inflammatory M1 and anti-inflammatory M2 phenotypes—directly determines the trajectory of central neuroinflammation and the chronification of pain (75). This framework is particularly relevant to JIA subtypes like oligoarticular and polyarticular forms, where spinal microglial activation correlates with persistent pain despite reduced joint inflammation (26, 76).

The interventional logic of acupuncture is profound because it does not target a single molecule but rather orchestrates a systemic reset of the entire dysregulated neuro-immune network. The elegance of its action lies in its utilization of the body’s innate somatosensory-autonomic reflex arc. By precisely stimulating peripheral somatosensory nerves, acupuncture can: peripherally, systemically suppress the body’s inflammatory response by activating the vagus nerve-cholinergic anti-inflammatory pathway; and centrally, directly modulate the functional programs of microglia. This modulation is achieved by inhibiting “danger signal” receptors on the microglial surface (e.g., TLR4, P2X7R) and their downstream pro-inflammatory signaling pathways (e.g., p38 MAPK, ERK), but it also fundamentally shifts microglia from “destroyers” (M1 type) to “restorers” (M2 type) while simultaneously resetting their mitochondrial metabolic homeostasis (via activation of the AMPK and Nrf2 pathways) and rewriting their gene expression “long-term memory” (through epigenetic mechanisms like DNA methylation, histone modification, and miRNAs) (13). In JIA-specific preclinical models (such as collagen-induced arthritis), this multi-target approach has been demonstrated to be effective, primarily by modulating inflammatory pathways like NLRP3/NF-κB (77). Furthermore, by inhibiting microglia, this approach is hypothesized to reduce the release of mediators like BDNF, which are known to cause the shift in neuronal anion gradients (GABAergic inhibition) that underlies central sensitization (77, 78). This systemic regulatory action perfectly answers the scientific questions posed in the introduction, providing a solid neuro-immunological foundation for understanding the treatment of JIA-associated pain with acupuncture.

### 6.2 Challenges and opportunities in translational medicine: the gap and bridge from mechanism to clinic

Although basic research has revealed the sophisticated mechanisms of neuro-immune modulation by acupuncture, successfully translating these findings into clinical practice remains a significant challenge. The core challenge is the lack of non-invasive biomarkers that can dynamically monitor central neuroinflammation and glial cell functional states in children with JIA. We cannot directly observe whether the AMPK or Nrf2 pathways in the spinal microglia of a child have been effectively activated. This “black box” scenario is a key bottleneck limiting the standardization and personalization of acupuncture therapy. Furthermore, the high degree of heterogeneity within JIA itself—spanning genetic, clinical, and inflammatory subtypes—along with the ethical and operational complexities of pediatric research, makes conducting large-scale, high-quality randomized controlled trials (RCTs) particularly difficult.

Limitations Key limitations of the current evidence base include significant protocol heterogeneity in acupuncture applications (e.g., manual acupuncture versus electroacupuncture; single-point stimulation at ST36 versus multi-acupoint combinations; variations in frequency and intensity that differentially impact outcomes such as visual analog scale (VAS) pain scores or cytokine levels); scarcity of high-quality pediatric RCTs (with only a few identified in recent meta-analyses, with variable outcomes like short-term pain relief versus long-term improvements in health-related quality of life (HRQoL)) (79, 80); limited generalizability across JIA subtypes (stronger evidence in oligoarticular JIA, with fewer studies on systemic or enthesitis-related forms); and ethical barriers to invasive CNS monitoring, such as lumbar punctures for direct glial biomarker assessment (81). Additionally, while electroacupuncture demonstrates superior vagal anti-inflammatory effects in preclinical models, its standardization remains inconsistent, potentially confounding clinical reproducibility. Future studies must address these gaps through protocol harmonization and subgroup analyses to enhance precision and applicability. Furthermore, it is critical to note that many of the specific molecular mechanisms discussed, particularly those in section 5.2 regarding mitochondrial homeostasis (e.g., PINK1/Parkin and PGC-1α pathways), are extrapolated from preclinical models of other diseases (such as Parkinson’s, stroke, or diabetes). Their direct validation within a JIA-specific pain context has not yet been established and remains a key direction for future research.

However, where there are challenges, there are also opportunities. The opportunity in translational medicine lies in developing accessible peripheral biomarkers that can reflect the state of the central neuro-immune system. A highly promising direction is the study of Extracellular Vesicles (EVs). EVs, particularly exosomes, are “messengers” for intercellular communication that can encapsulate proteins and nucleic acids (such as miRNAs) from their cell of origin and cross the blood-brain barrier into the peripheral circulation. In theory, by isolating EVs originating from the nervous system (e.g., from neurons or glial cells) in peripheral blood and analyzing their specific molecular cargo, it may be possible to “peek” into the changes occurring within the CNS. For example, after acupuncture treatment, changes in the levels of specific miRNAs (like miR-124, miR-146a) or proteins (like TREM2) inside EVs derived from microglia could serve as “liquid biopsy” indicators of the functional state of central microglia (2). This offers a potential bridge to connect basic mechanisms with clinical efficacy assessment, thereby enabling objective, quantitative monitoring of acupuncture’s effects and providing a basis for personalized treatment in JIA cohorts.

### 6.3 Future research directions and prospects for clinical translation

To advance the application of acupuncture in JIA pain management from “experience-based” to “evidence-based” and “precision” medicine, future research should prioritize parametric optimization through rigorously designed animal experiments that systematically compare different frequencies, intensities, and acupoint combinations of acupuncture stimulation and their differential modulation of p38/ERK signaling pathways, mitophagy levels, and epigenetic modifications within microglia (82). This will identify optimal parameter combinations that maximize anti-inflammatory and neuroprotective effects, providing a scientific foundation for standardizing clinical protocols. Concurrently, well-designed prospective clinical studies are essential to validate the correlation between potential biomarkers—such as specific miRNAs in peripheral blood extracellular vesicles—and improvements in pain scores and functional recovery in children with JIA, including the establishment of reference value ranges and assessments of sensitivity and specificity for predicting treatment response (83). Once validated, these biomarkers could underpin the development of detection kits to screen responsive patient populations, enabling truly personalized therapeutic approaches.

Advanced neuroimaging techniques should also be actively employed to visualize acupuncture’s central effects *in vivo*. For instance, positron emission tomography (PET) using tracers that specifically label activated microglia and reactive astrocytes—such as second-generation tracers targeting the translocator protein (TSPO) or P2X7 receptors—in PET-MR multimodal scans could quantitatively assess the inhibitory impact on neuroinflammation in the brain and spinal cord of JIA patients (84). This would yield the most direct human evidence for acupuncture’s mechanisms, bridging preclinical insights with clinical outcomes. Finally, clinical trials should explore multimodal therapy protocols that integrate acupuncture with modern disease-modifying antirheumatic drugs (DMARDs) or biologics alongside other non-pharmacological interventions like physical therapy and cognitive-behavioral therapy. The neuro-immune modulatory effects of acupuncture may synergize with pharmacological agents, enhancing patient adherence and outcomes by alleviating pain perception and psychological burden while minimizing drug dosages and associated side effects (79, 85). Through these multi-dimensional efforts, acupuncture can evolve into an indispensable, precision neuromodulatory tool, grounded in robust evidence, for the comprehensive management of chronic pain in children with JIA.

## 7 Conclusion

This review systematically demonstrates that the persistent pain associated with Juvenile Idiopathic Arthritis (JIA) is not merely a peripheral joint problem. Its core pathology lies within a complex neuro-immune dysregulation network that drives a central sensitization process, in which the aberrant activation of microglia serves as a critical node. This understanding elevates the research perspective on JIA pain from one of localized inflammation to the level of maladaptive remodeling of the central nervous system’s function and structure.

Building on this foundation, this paper has further elucidated the modern neuro-modulatory mechanism of acupuncture intervention. It is not a simple symptomatic analgesic but rather a systemic reset of the entire dysregulated network, achieved by activating the body’s innate “somatosensory-autonomic reflex.” At the molecular level, acupuncture systemically suppresses peripheral inflammation via the vagus nerve pathway and, more importantly, directly modulates the functional programs of central microglia. This modulation encompasses the inhibition of upstream pro-inflammatory signaling pathways (e.g., p38/ERK), the resetting of cellular mitochondrial metabolism and redox homeostasis, and the long-term reprogramming of gene expression through epigenetic mechanisms (e.g., DNA methylation, histone modification, and miRNAs). Ultimately, these actions reverse the functional phenotype of microglia from a “pro-inflammatory” M1 state to an “anti-inflammatory and reparative” M2 state.

Although significant progress has been made in mechanistic research, the core task for the future is “translation”—transforming these profound molecular discoveries into clinically applicable tools and strategies. This requires a dedicated effort to develop and validate peripheral biomarkers (such as specific miRNAs in circulating EVs) that reflect the central neuro-immune status and to visualize the central effects of acupuncture using advanced neuroimaging techniques (such as glia-targeting PET). The ultimate goal is to advance acupuncture into a predictable, quantifiable, and reproducible precision neuromodulatory therapy and to integrate it scientifically and rationally into modern multimodal treatment protocols for JIA, thereby providing a safer and more effective new avenue for pain management for affected children.
